# High Volumetric Energy Density Sulfur Cathode with Heavy and Catalytic Metal Oxide Host for Lithium–Sulfur Battery

**DOI:** 10.1002/advs.201903693

**Published:** 2020-05-06

**Authors:** Ya‐Tao Liu, Sheng Liu, Guo‐Ran Li, Tian‐Ying Yan, Xue‐Ping Gao

**Affiliations:** ^1^ Institute of New Energy Material Chemistry School of Materials Science and Engineering Renewable Energy Conversion and Storage Center Nankai University Tianjin 300350 China

**Keywords:** catalytic conversion, cathodes, heavy metal oxides, lithium–sulfur batteries, volumetric energy density

## Abstract

For high‐energy lithium–sulfur batteries, the poor volumetric energy density is a bottleneck as compared with lithium–ion batteries, due to the low density of both the sulfur active material and sulfur host. Herein, in order to enhance the volumetric energy density of sulfur cathode, a universal approach is proposed to fabricate a compact sulfur cathode with dense materials as sulfur host, instead of the old‐fashioned lightweight carbon nanomaterials. Based on this strategy, heavy lanthanum strontium manganese oxide (La_0.8_Sr_0.2_MnO_3_), with a high theoretical density of up to 6.5 g cm^−3^, is introduced as sulfur host. Meanwhile, the La_0.8_Sr_0.2_MnO_3_ host also acts as an efficient electrocatalyst to accelerate the diffusion, adsorption, and redox dynamics of lithium polysulfides in the charge–discharge processes. As a result, such S/La_0.8_Sr_0.2_MnO_3_ cathode presents high gravimetric/volumetric capacity and outstanding cycling stability. Moreover, an ultra‐high volumetric energy density of 2727 Wh L^−1^_‐cathode_ is achieved based on the densification effect with higher density (1.69 g cm^−3^), which is competitive to the Ni‐rich oxide cathode (1800–2160 Wh L^−1^) of lithium–ion batteries. The current study opens up a path for constructing high volumetric capacity sulfur cathode with heavy and catalytic host toward practical applications of lithium–sulfur batteries.

## Graphical abstract

Heavy metal oxides are more suitable than light carbon materials to fabricate compact cathode for lithium–sulfur batteries. Specifically, lanthanum strontium manganese oxide nanofibers, with the tap density of 2.59 g cm^−3^, display efficient catalytic activity toward lithium polysulfides, enhancing the volumetric energy density of sulfur cathode, which can even exceed lithium–ion batteries.

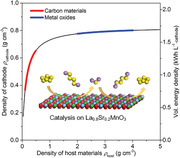

Achieving higher energy density is the continuous driving force for the development of secondary batteries. Among all the commercial secondary batteries, lithium‐ion batteries (LIBs) possess high gravimetric and volumetric energy densities, almost approaching their limitation of energy densities based on the inherent intercalation chemistry.^[^ ^1^ ^]^ Beyond LIBs, lithium–sulfur (Li‐S) battery has attracted considerable attention due to the high theoretical gravimetric and volumetric energy densities of 2600 Wh kg^−1^ and 2800 Wh L^−1^, respectively.^[^ ^2^ ^]^ Specifically, Li‐S battery has achieved a high gravimetric energy density of 350–609 Wh kg^−1^ in the past few years,^[^ ^3^ ^]^ showing a favorable application prospect in unmanned aerial vehicles.^[^ ^4^ ^]^ However, the corresponding volumetric energy density of Li‐S battery is limited within 325–581 Wh L^−1^ mainly due to the presence of the lightweight carbon nanomaterials as host or low sulfur content in sulfur cathode,^[^ ^3^, ^5^ ^]^ which is still far behind that of LIBs (670 Wh L^−1^ for LIBs).^[^ ^3b^ ^]^ For practical applications, the volumetric energy density should be considered simultaneously in the development of high‐energy Li‐S battery.

Intrinsically, the inferior volumetric energy density of Li‐S battery stems from the low mass density of sulfur (*ρ* = 2.07 g cm^−3^), in comparison to the heavy Li‐intercalation metal oxides as cathode in LIBs (e.g., 5.1 g cm^−3^ of LiCoO_2_). It means that it is very difficult to construct high mass density sulfur cathode (S‐cathode), which is further aggravated by the commonly used lightweight porous carbon nanomaterials as sulfur host.^[^ ^6^ ^]^ Therefore, it is impossible for the porous S‐cathode to compete against the metal oxide cathode of LIBs in terms of volumetric energy density (**Figure** **1**A). Theoretically, a feasible strategy for enhancing the volumetric energy density of Li‐S battery is to fabricate the compact S‐cathode, in which the mass density of the cathode should be increased to 1.34–1.61 g cm^−3^ in alignment with the state‐of‐the‐art LIBs, by assuming high sulfur content of 64 wt% and large discharge capacity of 1000 mAh g^−1^_‐sulfur_ in S‐cathode (Figure 1A). Recently, some important attempts have been made to increase the mass density of the S‐cathode, including high sulfur content,^[^ ^7^ ^]^ dense graphene host,^[^ ^8^ ^]^ compact electrode structure,^[^ ^9^ ^]^ and Mo_6_S_8_ and FeS as active materials.^[^ ^5^, ^10^ ^]^ However, these efforts still fail to meet the requirement on the basis of the gravimetric/volumetric capacity, and cycle stability. For example, the high volumetric energy density of 654 Wh L^−1^ can be obtained only for Li‐S primary battery, without consideration of the cycling feature.^[^ ^11^ ^]^ Although the freestanding matrix cathode could provide high areal capacity owing to the high sulfur loading without binder. However, the fluffy and porous features are unfavorable for high volumetric capacity of the S‐cathode.^[^ ^1^, ^12^ ^]^ Therefore, exploring a universal strategy to build compact S‐cathode is highly important in order to fulfill the practical applications of Li‐S battery in future.

**Figure 1:**
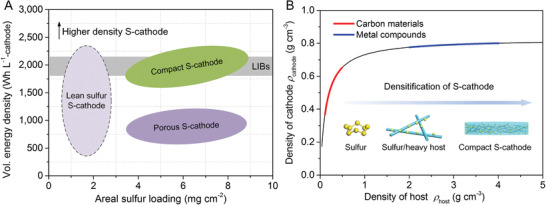
Scheme of the volumetric energy density in Li‐S battery. A) Projected volumetric energy density of various S‐cathodes as a function of areal sulfur loading. This scheme is based on the previous literatures in Table S5 (Supporting Information), in which the volumetric energy density is between 1800 and 2160 Wh L^−1^ for the Ni‐rich oxide cathode of LIBs.^[^ ^3b^ ^]^ B) Plot of mass density of S‐cathodes against host density. The derivation of mathematical function of the cathode density against host density is available in the Supporting Information. The inset shows the densification of the S‐cathode through heavy host materials.

How to fabricate the desirable compact S‐cathode? A three “high” principle is proposed in this study to fabricate compact S‐cathode for achieving high volumetric energy density (Figure 1): (1) “high” sulfur content of above 80 wt% for the sulfur‐based composite, (2) “high” sulfur loading of above 4 mg cm^−2^ for thick sulfur cathode, and (3) “high” density for the host materials (heavy host) with good electrocatalytic activity. Herein, heavy metal cobaltites/ferrites are demonstrated to be effective to fabricate porous sulfur‐based composites with high tap density,^[^ ^13^ ^]^ which is the basic element for compact S‐cathode. For example, when lithium cobalt oxide and carbon nanotubes with obviously different densities are used as hosts, the S‐cathode densities are estimated to be 0.80 and 0.49 g cm^−3^, respectively (details are provided in the Supporting Information). It means that the density of the host materials should be as high as possible to increase the tap density of the sulfur‐based composites according to the densification effect of the core components (sulfur and host materials). Besides, host materials are required to be electrocatalytic for accelerating the conversion of soluble intermediate lithium polysulfides (LiPS) in the charge–discharge processes, in order to ensure high sulfur utilization, good cycle stability, and desired energy density output.

As a proof of the three “high” principle, lanthanum strontium manganese oxide (La_0.8_Sr_0.2_MnO_3_; theoretical density of 6.5 g cm^−3^),^[^ ^14^ ^]^ which is a well‐known electrocatalyst for oxygen reduction,^[^ ^15^ ^]^ is adopted as host material. With heavy metal oxide nanofibers, we can build a compact S‐cathode with high mass density of up to 1.69 g cm^−3^, and high sulfur content of around 65 wt% in the resultant S‐cathode. Furthermore, the unique 1D nanofiber morphology and perovskite structure can provide sufficient active sites for the catalytic conversion of LiPS, endowing the cathode with a large reversible capacity and good cycle stability. Remarkably, the compact S/La_0.8_Sr_0.2_MnO_3_ cathode could achieve an ultra‐high volumetric energy density of 2727 Wh L^−1^_‐cathode_ with a high sulfur loading of 6.2 mg cm^−2^, outperforming the 1308 Wh L^−1^_‐cathode_ of the conventional porous S/carbon cathode, as well as the best metal oxide cathode of LIBs (1800–2160 Wh L^−1^ for Panasonic NCR18650B cathode).

La_0.8_Sr_0.2_MnO_3_ nanofibers are prepared by an electrospinning–calcination method (details are provided in the Experimental Section). Under the optimized condition, the nanofibers have a uniform diameter of ≈230 nm with a length of tens of micrometers (**Figure** **2**A,B and Figure S1, Supporting Information). The nanofibers are composed of irregular secondary nanocrystals, with rough surface and small voids filling inside the fiber. Such 1D structure ensures sufficient contact between the host and sulfur, facilitating the surface catalysis of soluble LiPS in the charge–discharge processes. High‐resolution transmission electron microscopy (HRTEM) image in Figure 2C displays the lattice fringe of 0.28 nm, corresponding to the dominant exposed (110) facet of La_0.8_Sr_0.2_MnO_3_. The diffraction rings in the selected area electron diffraction (SAED) pattern (Figure 2C, inset) agree well with X‐ray diffraction (XRD) pattern in Figure 2D, indicating good polycrystallinity of La_0.8_Sr_0.2_MnO_3_. In the crystallographic structure, La_0.8_Sr_0.2_MnO_3_ is assigned to the rhombohedral perovskite structure (JCPDS 53‐0058). N_2_ sorption in Figure 2E reveals that the nanofibers have a specific surface area of 131 m^2^ g^−1^, with dominant mesopores of ≈2.1 nm. The S/La_0.8_Sr_0.2_MnO_3_ composite is prepared using the typical melt–evaporation method, by heating the nanofibers and sulfur at 155 °C and further up to 300 °C. The sulfur content in the S/La_0.8_Sr_0.2_MnO_3_ composite is 81 wt% (Figure 2F), where the high sulfur coverage results in the sharp decline of surface area of the composite down to 32 m^2^ g^−1^ (Figure S2, Supporting Information). The energy dispersive spectroscopy indicates that sulfur is uniformly dispersed along the nanofiber, altogether with the coexisting of La, Sr, Mn, and O elements (Figure S3, Supporting Information). In this work, the aligned carbon nanotubes (A‐CNTs) with 1D structure are selected as the contrast sample. The A‐CNTs have a length of tens of micrometers, and a high specific surface area of 395 m^2^ g^−1^, with mesoporous size of ≈2.3 nm (Figures S4 and S5, Supporting Information).

**Figure 2:**
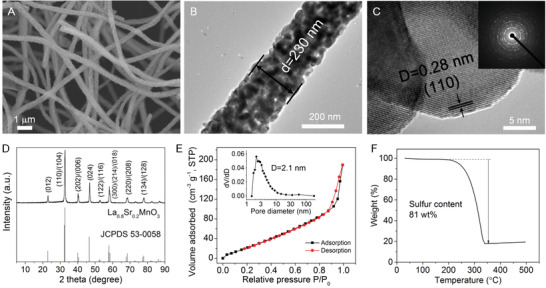
Characterizations of La_0.8_Sr_0.2_MnO_3_ nanofibers and S/La_0.8_Sr_0.2_MnO_3_ composite. A) Scanning electron microscope (SEM) image of La_0.8_Sr_0.2_MnO_3_ nanofibers. B) Transmission electron microscope (TEM) image of La_0.8_Sr_0.2_MnO_3_ nanofibers. C) HRTEM image and the (inset) SAED pattern of La_0.8_Sr_0.2_MnO_3_ nanofibers. D) XRD pattern of La_0.8_Sr_0.2_MnO_3_ nanofibers. E) N_2_ sorption isotherm and pore distribution of La_0.8_Sr_0.2_MnO_3_ nanofibers. F) Thermogravimetric (TG) curve of the S/La_0.8_Sr_0.2_MnO_3_ composite in Ar atmosphere.

La_0.8_Sr_0.2_MnO_3_ is electrochemically stable in the Li‐S battery operation window (Figure S6, Supporting Information). In this study, the feasibility as sulfur host is evaluated by the capacity of the S‐cathode to emphasize the overall energy density of the whole electrode, rather than only the active sulfur or S‐composite. The S/La_0.8_Sr_0.2_MnO_3_ presents typical discharge potential profiles with two plateaus, manifesting the stepwise redox behavior of sulfur in the ether‐based electrolyte. At a low sulfur loading, both S/La_0.8_Sr_0.2_MnO_3_ and S/A‐CNT cathodes exhibit comparable capacity at various C‐rates, with the former displaying a better cycling stability at C/2 rate in the flooded electrolyte of10 µL mg^−1^ (Figure S7a–c, Supporting Information). In the lean electrolyte of 5 µL mg^−1^, however, the S/A‐CNT suffers from a severe potential hysteresis of 0.2 V at C/10 rate. Meanwhile, the S/La_0.8_Sr_0.2_MnO_3_ displays superior 792 mAh g^−1^_‐cathode_ with a small potential hysteresis of 0.13 V (**Figure** **3**A) and much better cycling performance with 532 mAh g^−1^_‐cathode_ after 150 cycles (Figure 3B). Furthermore, in the leaner electrolyte of 4 µL mg^−1^, the S/La_0.8_Sr_0.2_MnO_3_ still maintains a satisfactory capacity and capacity retention, while the S/A‐CNT cannot functionalize properly (Figure S7d, Supporting Information). Thus, La_0.8_Sr_0.2_MnO_3_ is an effective host to catalyze the redox reactions of sulfur, even in the severe environment of high viscosity and sluggish redox kinetics with lean electrolyte,^[^ ^16^ ^]^ which is important for high energy density of Li‐S battery. The S/La_0.8_Sr_0.2_MnO_3_ displays a good rate performance from C/10 rate to 2C rate at the electrolyte/sulfur (E/S) ratio of 15 µL mg^−1^ and from 0.25 to 2 mA cm^−2^ at the E/S ratio of 7 µL mg^−1^ (Figure S8, Supporting Information), indicating that the high rate capability of the S/oxide composites is dominated by the redox kinetics, rather than electronic conductivity of sulfur hosts.^[^ ^13^ ^]^

**Figure 3:**
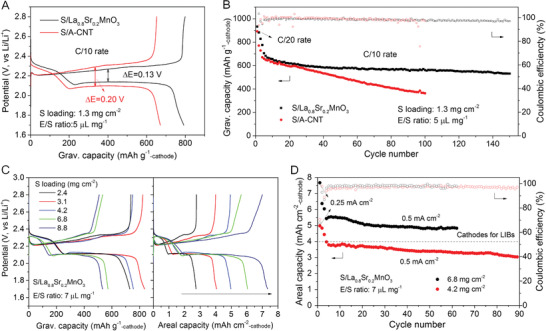
Electrochemical performance of the S/La_0.8_Sr_0.2_MnO_3_ cathode. A) Potential profiles and B) cycling curves of S/La_0.8_Sr_0.2_MnO_3_ and S/A‐CNT cathodes in 5 µL mg^−1^ electrolyte at sulfur loading of 1.3 mg cm^−2^. C) Potential profiles as gravimetric and areal capacity of S/La_0.8_Sr_0.2_MnO_3_ cathode in 7 µL mg^−1^ electrolyte at various sulfur loadings. The current densities are 0.3, 0.23, 0.25, 0.25, and 0.66 mA cm^−2^, respectively, for the cathodes with different sulfur loading of 2.4, 3.1, 4.2, 6.8, and 8.8 mg cm^−2^. D) Cycling curves as areal capacity of S/La_0.8_Sr_0.2_MnO_3_ cathode in 7 µL mg^−1^ electrolyte.

Next, the performance of the S/La_0.8_Sr_0.2_MnO_3_ cathode with high sulfur loadings is evaluated, which is essential for practical application.^[^ ^3^, ^17^ ^]^ At the sulfur loading of 2.4 mg cm^−2^, the S/La_0.8_Sr_0.2_MnO_3_ delivers high gravimetric capacities of 729 and 610 mAh g^−1^_‐cathode_ at 0.3 and 0.6 mA cm^−2^, respectively, with an outstanding cycling performance and high coulombic efficiency of 98% over 210 cycles, superior to the S/A‐CNT (Figure S9, Supporting Information). Further, when the sulfur loading is increased to 3.1, 4.2, 6.8, and 8.8 mg cm^−2^, the S/La_0.8_Sr_0.2_MnO_3_ delivers 834, 755, 572, and 533 mAh g^−1^_‐cathode_ at a low current density, respectively, corresponding to the areal capacity of 4.1, 5.0, 6.0, and 7.3 mAh cm^−2^_‐cathode_ (Figure 3C). At 4.2 mg cm^−2^ sulfur loading, the S/La_0.8_Sr_0.2_MnO_3_ exhibits a decent cycling stability, with a coulombic efficiency of around 97% during 89 cycles. In particular, the areal capacity of the S/La_0.8_Sr_0.2_MnO_3_ cathode outperforms the metal oxide cathode of conventional LIBs (4 mAh cm^−2^). After activation at the low density of 0.25 mA cm^−2^ for two cycles, the S/La_0.8_Sr_0.2_MnO_3_ cathode with the high sulfur loading of 6.8 mg cm^−2^ maintains large areal capacity and good cycling within 60 cycles, with a coulombic efficiency of around 98% (Figure 3D). Thus, the thick S‐cathode with high sulfur loadings can work in lean electrolyte (<10 µL mg^−1^) with the high areal capacity and good cycle performance, thanks to the unique 1D structure for easy penetration/diffusion of the electrolytes, as well as good electrocatalytic activity for the electrochemical conversion of the soluble LiPS. Previous study by McCloskey pointed out that an E/S ratio of 2 µL mg^−1^ or less should be required for Li‐S battery to fulfill the potential energy density.^[^ ^18^ ^]^ For evaluation in coin cells, the electrolyte usage is larger obviously as compared to that in full cells, due to the large dead volume in coin cells with less active materials. Here, the minimum E/S ratio for the thick S/La_0.8_Sr_0.2_MnO_3_ cathode is 5 µL mg^−1^ (Figure S7e,f, Supporting Information), which could be further reduced in the pouch cell with more active materials after structure optimization. What is more, the efficient and catalytic S/La_0.8_Sr_0.2_MnO_3_ cathode helps to protect the lithium anode, as evidenced by minor cracks and less LiS_2_/Li_2_S deposition on lithium surface (Figure S10, Supporting Information).

In this section, we focus on the superiority of the heavy La_0.8_Sr_0.2_MnO_3_ host in constructing high volumetric energy density S‐cathode (including S‐composite, binder, and conductive agent). First, heavy metal oxides have the obvious advantage to fabricate S‐based composites with a high tap density.^[^ ^13^, ^19^ ^]^ In this work, the measured tap density of the S/La_0.8_Sr_0.2_MnO_3_ composite is 1.61 g cm^−3^, much higher than that (0.92 g cm^−3^) of the S/A‐CNT composite. As a result, the S/La_0.8_Sr_0.2_MnO_3_ cathode, after mixing with conductive agent and binder, has a desired high density (0.98 g cm^−3^), almost double of that (0.45 g cm^−3^) for the S/A‐CNT cathode. The morphological change can be visually observed from top surface and cross‐section of the compact S/La_0.8_Sr_0.2_MnO_3_ cathode, and the loose S/A‐CNT cathode (**Figure** **4**A–H). Specifically, at the same sulfur loading of 6.2 mg cm^−2^, the thickness of the bare S/La_0.8_Sr_0.2_MnO_3_ cathode (94 µm) is much smaller than that of the bare S/A‐CNT cathode (208 µm) (Figure 4B,F). Correspondingly, the volumetric energy density of the bare S/La_0.8_Sr_0.2_MnO_3_ cathode of 1787 Wh L^−1^_‐cathode_ at 0.25 mA cm^−2^ is obtained, which is much higher than that of the bare S/A‐CNT cathode (689 Wh L^−1^_‐cathode_) (Figure 4J and Figure S11a, Supporting Information). More importantly, the cathode density could be increased further by pressing the cathode under a proper pressure of ≈0.2 MPa (Figure 4I), and thus further boosting the volumetric energy density (Figure 4J and Figure S11b, Supporting Information). Of course, higher press pressure of 0.5 MPa could decrease the porosity of the cathode, blocking the electrolyte penetration and severely harming the electrochemical performance (Figure S11C, Supporting Information). Under appropriate pressing, both cathodes show significantly enhanced density, with a compact surface, reduced thickness (Figure 4C,D,G,H), and low porosity (Table S4, Supporting Information), and the S/La_0.8_Sr_0.2_MnO_3_ cathode still exhibits higher density than S/A‐CNT cathode owing to the densification effect with heavy host feature. The volumetric energy density of S/La_0.8_Sr_0.2_MnO_3_ cathode reaches up to 2727 and 1779 Wh L^−1^_‐cathode_ at 0.25 and 0.5 mA cm^−2^, respectively, outdistancing those of S/A‐CNT cathode (1308 and 1033 Wh L^−1^_‐cathode_, respectively). Meanwhile, the optimized gravimetric energy density is also achieved in the S/La_0.8_Sr_0.2_MnO_3_ cathode (Figure S12, Supporting Information), demonstrating the unique superiority of La_0.8_Sr_0.2_MnO_3_ host to A‐CNT host in fabricating high energy density S‐cathode. Impressively, the pressed S/La_0.8_Sr_0.2_MnO_3_ cathode (4 mg cm^−2^ sulfur loading) displays an excellent cycling performance for 100 cycles, with an initial high volumetric energy density of 1917 Wh L^−1^_‐cathode_ and gravimetric energy density of 1038 Wh kg^−1^_‐cathode_ at 0.47 mA cm^−2^after initial activation (Figure 4K). The coulombic efficiency of the pressed S/La_0.8_Sr_0.2_MnO_3_ cathode is further improved, which is floated around 100% and then stabilized at 99% during 100 cycles (Figure S13a, Supporting Information).

**Figure 4:**
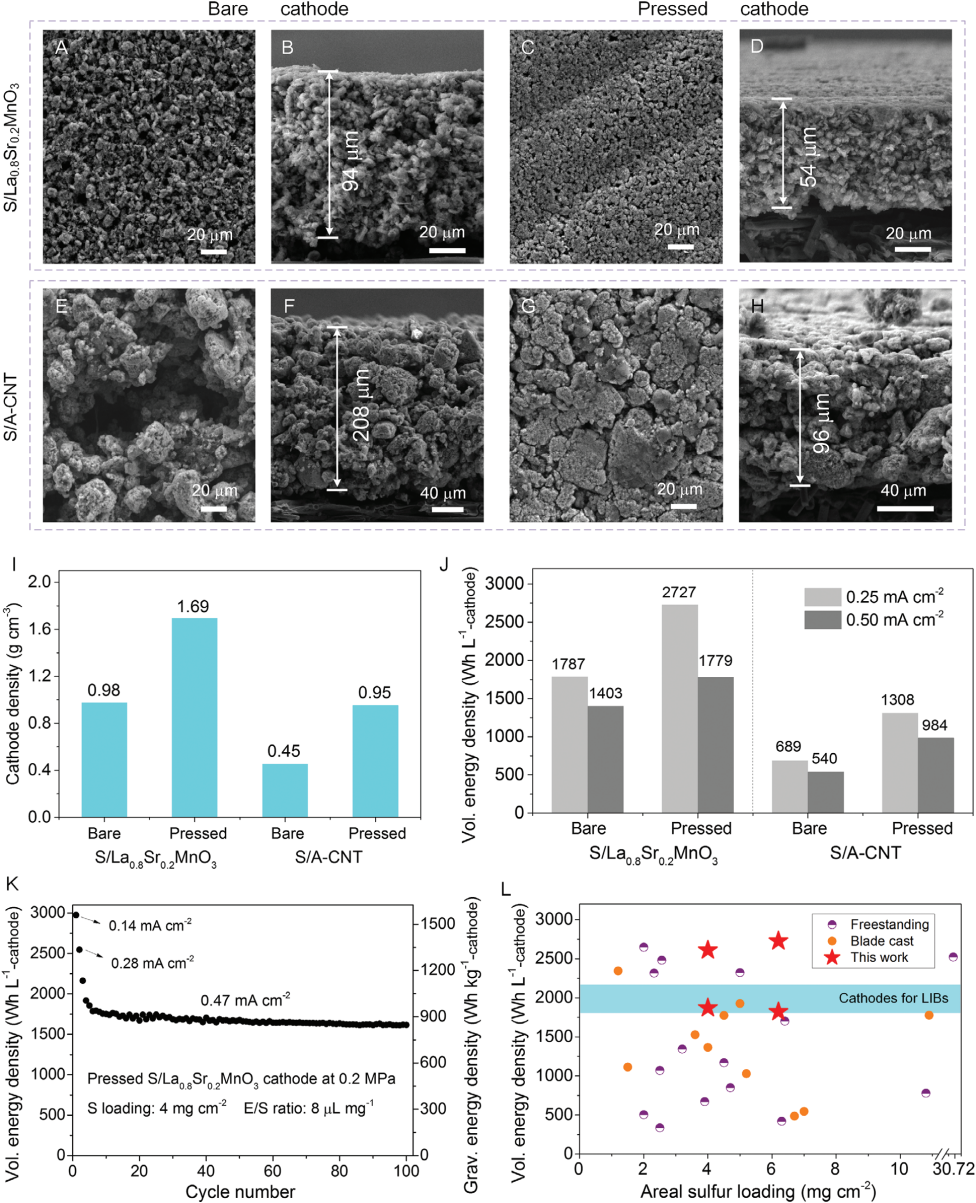
Volumetric energy density of the S/La_0.8_Sr_0.2_MnO_3_ and S/A‐CNT cathodes. SEM images of A) surface and B) cross‐section of bare S/La_0.8_Sr_0.2_MnO_3_ cathode, and C) surface and D) cross‐section of pressed S/La_0.8_Sr_0.2_MnO_3_ cathode. SEM images of E) surface and F) cross‐section of bare S/A‐CNT cathode, and G) surface and H) cross‐section of pressed S/A‐CNT cathode. I) Density and J) volumetric energy density of S/La_0.8_Sr_0.2_MnO_3_ and S/A‐CNT cathodes before and after pressing at 0.2 MPa. Cathode refers to the sulfur‐based composite, conductive agent, and binder, without current collector. K) Cycling stability of the S/La_0.8_Sr_0.2_MnO_3_ cathode after pressing at 0.2 MPa. L) Summary of the volumetric capacity of the various cathodes in literatures. The orange solid circles represent the traditional blade cast cathode, and the purple half‐filled circles represent the freestanding cathode (Table S5, Supporting Information).

Figure 4L summarizes the volumetric energy density of various S‐cathodes reported in literatures (Table S5, Supporting Information), with a focus on carbon nanomaterials or hybrids as host materials. As shown in Figure 4L, the compact S/La_0.8_Sr_0.2_MnO_3_ cathode presents outstanding volumetric energy density as compared with previous studies, including carbon, metal sulfides,^[^ ^5^, ^20^ ^]^ and oxides^[^ ^21^ ^]^ as host materials. In particular, the volumetric energy density of the cathode at 0.25 mA cm^−2^ exceeds that of the Ni‐rich metal oxide cathode of LIBs. Thus, we demonstrate in this study that Li‐S battery could compete against LIBs in terms of volumetric energy density, with optimization of the compact S‐cathode preparation technology. Despite the high energy density can be achieved, it should be noted that the Li‐S battery lifetime still pales in comparison to the ultra‐long cycling of state‐of‐the‐art LIBs, and therefore more effort is needed to tackle this challenge.

It is worth noting that the resultant low porosity by pressing the cathode is in favor of further reducing the electrolyte usage (Figure S14, Supporting Information). For the pressed S/La_0.8_Sr_0.2_MnO_3_ cathode, the high capacity output and good cycling are obtained under 6 µL mg^−1^ electrolyte, superior to 7 µL mg^−1^ for bare cathode (Figure 3). Specifically, the E/S ratio in coin cells could be decreased to a minimum of 4 µL mg^−1^, which is a much desirable value to boost the energy density of full cells. Nevertheless, with such a low electrolyte, a large capacity irreversibility appears in the initial charge process and the discharge plateau suffers an obvious polarization, possibly due to the inhomogeneous Li_2_S/Li_2_S_2_ deposition or the limited lithium–ion transportation. Therefore, the cathode structure still needs to be optimized to fulfill its potential.

Intrinsically, the diffusion, adsorption, and charge‐transfer processes are involved in the electrochemical dissolution/deposition reaction of sulfur cathode, with the soluble LiPS as intermediate from insoluble sulfur to insoluble Li_2_S.^[^ ^13a^ ^]^ In particular, the electrocatalytic conversion of the soluble LiPS is the key issue for ensuring the cycle stability of the S‐cathode. From cyclic voltammograms (CVs) (**Figure** **5**A,B), sulfur undergoes better reversibility and smaller polarization on La_0.8_Sr_0.2_MnO_3_ host, indicating the good electrocatalytic activity of La_0.8_Sr_0.2_MnO_3_, which is considered as the intrinsic driving force to stimulate the S‐cathode electroactivity.^[^ ^22^ ^]^ To elucidate the mechanism of the electrocatalytic effect, the diffusion, adsorption, and charge‐transfer processes of the soluble LiPS on the electrode/electrolyte interface are further detected.

**Figure 5:**
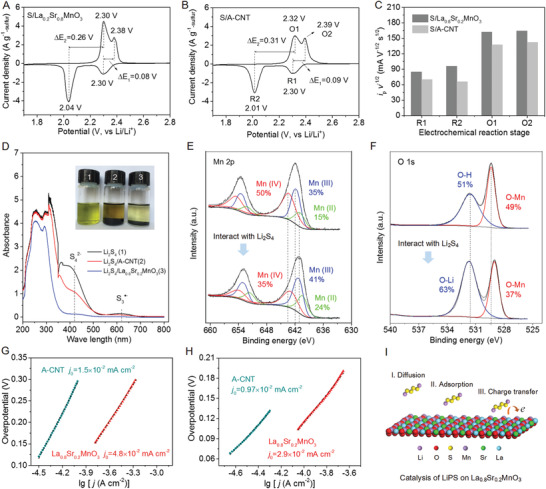
Mechanism of the catalytic conversion of LiPS on La_0.8_Sr_0.2_MnO_3_. A–C) Diffusion of LiPS on La_0.8_Sr_0.2_MnO_3_. CVs of A) S/La_0.8_Sr_0.2_MnO_3_ and B) S/A‐CNT cathodes at 0.1 mV s^−1^. C) Slope of *i*_p_/*v* ^1/2^, where *i*_p_ and *v* refer to the peak current and scan rate of the CVs, respectively (Figures S15 and S16, Supporting Information). (R1: S_8_+*e*→S*_x_* ^2−^; R2: Li^+^+S*_x_* ^2−^+*e*→Li_2_S_2_/Li_2_S; O1: Li_2_S_2_/Li_2_S→Li^+^+S*_x_* ^2−^+*e*; O2: S*_x_* ^2−^→S_8_+*e* (4≤*x*≤8)). D–F) Adsorption of LiPS on La_0.8_Sr_0.2_MnO_3_ and the chemical interaction characterized by XPS. D) Visual observation of Li_2_S_4_ adsorption on La_0.8_Sr_0.2_MnO_3_: photos of Li_2_S_4_ adsorption test and UV–vis spectra of the supernatant. E) Mn 2p core level of (up) pristine La_0.8_Sr_0.2_MnO_3_ and (down) Li_2_S_4_/La_0.8_Sr_0.2_MnO_3_. F) O1s core level of (up) pristine La_0.8_Sr_0.2_MnO_3_ and (down) Li_2_S_4_/La_0.8_Sr_0.2_MnO_3_. G,H) Charge transfer of LiPS. Tafel plots of La_0.8_Sr_0.2_MnO_3_ and A‐CNT electrodes derived from current density–voltage (*J*–*V*) curves in the G) cathodic and H) anodic process. *J*–*V* curves are available in Figure S20 (Supporting Information). I) Schematic illustration of the mechanism of the electrocatalysis of LiPS on La_0.8_Sr_0.2_MnO_3_, in successive diffusion, adsorption, and charge‐transfer processes.

The diffusion process is characterized by CVs at various scan rates from 0.1 to 0.5 mV s^−1^ (Figure S15 and Equation (S10), Supporting Information). Typically, the peak current has a linear relationship with the square root of scan rate for the stepwise reduction and oxidation (Figure S16, Supporting Information), suggesting that the rate determining step is dominated by the diffusion of soluble LiPS.^[^ ^23^ ^]^ Moreover, the faster diffusion of soluble LiPS in the multistep reaction processes is verified based on the relatively larger plot slopes (*i*_p_/*v* ^1/2^) (Figure 5C), which is originated from the strong chemical interaction between soluble LiPS and polar La_0.8_Sr_0.2_MnO_3_ nanofibers.

The adsorption is identified by the visual observation test by choosing Li_2_S_4_ as the representative of LiPS. After the settlement observation for 24 h, the color of the Li_2_S_4_ solution containing La_0.8_Sr_0.2_MnO_3_ nanofibers is much lighter (Figure 5D), and the absorbance of characteristic S_4_^2−^ peak at 410 nm in UV–vis spectra of the solution almost disappears,^[^ ^24^ ^]^ demonstrating the strong adsorption of the soluble LiPS on the polar La_0.8_Sr_0.2_MnO_3_ nanofibers. Further, the adsorption and interaction of Li_2_S_4_ on La_0.8_Sr_0.2_MnO_3_ nanofibers are analyzed by X‐ray photoelectron spectra (XPS). The Li_2_S_4_/La_0.8_Sr_0.2_MnO_3_ exhibits four sulfur environments, i.e., the terminal sulfur (S_T_^−1^) at 161.6 eV, bridging sulfur (S_B_^−1^) at 163.3 eV of Li_2_S_4_, high valence state of thiosulfate at 166.7 eV, and polythionate complex at168.7 eV (Figure S17, Supporting Information).^[^ ^25^ ^]^ Correspondingly, the chemical state of Mn 2p core level of La_0.8_Sr_0.2_MnO_3_ is varied dramatically (Figure 5E). After interacting with Li_2_S_4_, the fraction of Mn (IV) decreases sharply from 50% to 35%, accompanied by the increase of both Mn (II) and Mn (III), suggesting that Li_2_S_4_ can be oxidized partially by Mn (IV) to thiosulfate and polythionate complex as efficient mediators.^[^ ^26^ ^]^ Besides, the binding energy of Mn 2p shifts negatively by 0.3–0.6 eV, indicating a strong binding between Mn and S atoms. On the O 1s core level, the O‐Li binding appears at 531.2 eV in Li_2_S_4_/La_0.8_Sr_0.2_MnO_3_, which simultaneously brings more electron density to negatively shift the O‐Mn binding (Figure 5F).^[^ ^27^ ^]^ The strong adsorption and chemical interaction between soluble LiPS and polar La_0.8_Sr_0.2_MnO_3_ nanofibers could accelerate the subsequent charge‐transfer process.

The charge‐transfer process is the critical step for the catalytic conversion of the soluble LiPS on La_0.8_Sr_0.2_MnO_3_ nanofibers in the charge–discharge processes.^[^ ^28^ ^]^ The electrochemical surface area (ECSA) of the working electrode is determined by CVs (Figures S18 and S19, Supporting Information),^[^ ^29^ ^]^ and the charge‐transfer reaction of LiPS is detected by the exchange current density (*j*_0_) from linear sweep voltammetry (LSV). Clearly, a remarkably higher current density can be found for the La_0.8_Sr_0.2_MnO_3_ electrode in both cathodic and anodic polarization (Figure S20, Supporting Information). In particular, the derived *j*_0_ from Tafel plot for La_0.8_Sr_0.2_MnO_3_ is 4.8 × 10^−2^ and 2.9 × 10^−2 ^mA cm^−2^ for the cathodic and anodic processes (Figure 5G), respectively, demonstrating the accelerated conversion of LiPS on La_0.8_Sr_0.2_MnO_3_. In situ electrochemical impedance spectra are conducted to further investigate the electrochemical reaction processes on electrolyte/electrode interface (Figure S21, Supporting Information). Briefly, the small charge‐transfer resistance, adsorption, and diffusion impedances at various depth of discharge and stage of charge are observed for the S/La_0.8_Sr_0.2_MnO_3_. Therefore, La_0.8_Sr_0.2_MnO_3_ can serve as efficient electrocatalyst and host, based on the evaluation of the diffusion, adsorption, and charge‐transfer processes of intermediate LiPS (Figure 5I).

It is concluded tentatively from the above discussion that the primary advantage of La_0.8_Sr_0.2_MnO_3_ is originated from its high density and catalytic activity, which could be considered as a prerequisite condition for host selection in terms of enhancing the volumetric energy density of sulfur cathode. Besides the electronic conductivity, morphology should also be optimized for better electrochemical performance. Moreover, the energy density might be further improved if the heavy host itself could contribute some capacity.^[^ ^5^ ^]^

In conclusion, we propose a universal approach for constructing highly compact S‐cathode on the basis of the three “high” principle and densification effect with heavy host materials. Specifically, after introducing the heavy La_0.8_Sr_0.2_MnO_3_ nanofibers, the sulfur cathode possesses a high density of 1.69 mg cm^−2^. Moreover, La_0.8_Sr_0.2_MnO_3_ nanofibers can serve as an efficient electrocatalyst to remarkably accelerate the diffusion, adsorption, and charge‐transfer processes of the intermediate soluble LiPS, enabling the excellent electrochemical performance of the S‐cathode. As a result, the compact S‐cathode exhibits both higher gravimetric capacity and volumetric capacity, along with good cycling stability. In particular, the S/La_0.8_Sr_0.2_MnO_3_ cathode can deliver an ultra‐high volumetric energy density of 2727 Wh L^−1^_‐cathode_ with the sulfur loading of 6.2 mg cm^−2^, exceeding the best intercalation oxide cathode of LIBs championship (Panasonic NCR18650B). Moreover, the low porosity of the compact S‐cathode could further decrease to electrolyte usage, in favor of enhancing the gravimetric energy density of full cells. We hope this work could ignite the passion for exploring more efficient heavy and catalytic host of sulfur to enhance the volumetric energy density of Li‐S battery for future application.

## Experimental Section

##### Preparation of La_0.8_Sr_0.2_MnO_3_ Nanofibers and S/La_0.8_Sr_0.2_MnO_3_ Composite

La_0.8_Sr_0.2_MnO_3_ nanofibers were prepared by electrospinning the solution of *N*,*N*‐dimethylformamide, polyacrylonitrile, lanthanum nitrate, strontium nitrate, and manganese acetate, and then by calcining the precursor fibers at 650 °C for 3 h in air. The flow rate in the electrospinning was 20 µL min^−1^, with the distance and voltage between the nozzle and collector being 12 cm and 15 kV, respectively. The A‐CNT was purchased from Nanjing XFNANO Materials Tech. Co., Ltd. The S‐based composites were prepared by heating the S/La_0.8_Sr_0.2_MnO_3_ or S/A‐CNT in Ar atmosphere at 155 °C for 6 h, and then at 300 °C for 3 h.

##### Characterization

Scanning electron microscope (SEM) (JEOL, JSM‐7800F) and TEM (JEOL, JEM‐2800) were used to characterize the morphology. XRD (Rigaku mini FlexII) was conducted to examine the crystallographic structure. XPS (Thermo Scientific ESCALAB 250Xi) was performed to investigate the chemical state of elements. Thermogravimetric (TG) analysis (METTLER TOLEDO, TGA/DSC1) was conducted to determine the sulfur content. The Brunauer–Emmett–Teller method (JW‐BK112 system) was applied to determine the surface area and pore distribution.

##### Electrochemical Measurement

The S‐cathodes were prepared by casting a slurry of S‐composite, conductive agent, and binder (mass ratio of 8:1.3:0.7) on current collectors. The conductive agent included A‐CNT and graphene and the binder was composed of carboxymethyl cellulose sodium (60 wt%) and styrene butadiene rubber (40 wt%). The diameters for thin and thick cathodes were 10 and 12 mm, respectively. The electrochemical performance was evaluated by assembling CR2032 coin cells, using S‐cathode, lithium anode, and Celgard 2300 separator. The electrolyte was prepared by dissolving lithium bis(trifluoromethanesulfonyl)imide (1 m) and LiNO_3_ additive (2 wt%) in 1,3‐dioxolane (DOL) and 1,2‐dimethoxyethane (DME) (1: 1, v/v). The charge/discharge test was conducted on LAND‐CT2001A instruments (1C = 1675 mA g^−1^) and the CV measurement was performed on a CHI 600e electrochemical station.

##### Adsorption of Li_2_S_4_

Li_2_S_4_ solution (2 mm L^−1^) was prepared by adding Li_2_S and S_8_ with a stoichiometric ratio in DME under vigorous stirring overnight at 60 °C, followed by adding La_0.8_Sr_0.2_MnO_3_ or A‐CNT (50 mg). After stabilizing for 24 h, the supernatant was taken out for photos and UV–vis absorption test (UV–vis, Varian Cary 100 Conc), while the precipitate was dried in Ar‐filled glove box for further XPS analysis.

##### Exchange Current Density (*j*_0_)

The ECSA was first obtained using a working electrode (La_0.8_Sr_0.2_MnO_3_ (LSMO) or A‐CNT), a platinum counter electrode, and a Ag/AgCl reference electrode in a three‐electrode electrochemical cell with 5 mm K_3_[Fe(CN)_6_] in 1 m KCl solution as electrolyte. CV scans from 10 to 100 mV s^−1^ were carried out in a potential window of 0–0.6 V (vs Ag/AgCl). The ECSA could be calculated by Equation (S10) of the Supporting Information, knowing that the diffusion coefficient of 5 mm K_3_[Fe(CN)_6_] in 1 m KCl was 0.76 × 10^−5^ cm^2^ s^−1^. The *j*_0_ was derived from the Tafel plot of LSV curves by selecting La_0.8_Sr_0.2_MnO_3_ (LSMO) or A‐CNT as the working electrode, and lithium foil as the counter and reference electrode in a three‐electrode electrochemical cell, with 30 × 10^−3^ m Li_2_S_4_ and 1 m lithium bis(trifluoromethanesulfonyl)imide (LiTFSI) in DME as electrolyte.

## Conflict of Interest

The authors declare no conflict of interest.

## Supporting information

Supporting Information
