# Supplementary material for: High Volumetric Energy Density Sulfur Cathode with Heavy and Catalytic Metal Oxide Host for Lithium–Sulfur Battery
Source: Adv Sci (Weinh). 2020 May 6;7(12):1903693. doi: 10.1002/advs.201903693 (PMC7312450; doi:10.1002/advs.201903693)
Supplement: Supplementary file 1 — Supporting Information [file ADVS-7-1903693-s001.pdf]

Supporting Information

High Volumetric Energy Density Sulfur Cathode with Heavy and Catalytic Metal Oxide Host for Lithium-Sulfur Battery

*Ya-Tao Liu, Sheng Liu, Guo-Ran Li, Tian-Ying Yan, Xue-Ping Gao**

Institute of New Energy Material Chemistry, School of Materials Science and Engineering, Renewable Energy Conversion and Storage Center, Nankai University, Tianjin 300350, China.

E-mail: xpgao@nankai.edu.cn

Evaluation of the tap density of S-cathode.

Table S1.Four components in sulfur cathode and their physical feature.

Characteristics	Component 1 (sulfur)	Component 2 (Host material)	Component 3 (Conductive agent)	Component 4 (Binder)
Tap density/g cm^{-3}	ρ_1	ρ_2	ρ_3	ρ_4
Mass fraction/wt%	w_1	w_2	w_3	w_4
Volume Fraction/%	v_1	v_2	v_3	v_4

$$\begin{cases} w_1 = \dfrac{\rho_1(1-v_2-v_3-v_4)}{\rho_1(1-v_2-v_3-v_4)+\rho_2 v_2+\rho_3 v_3+\rho_4 v_4} \text{(Equation S1)} \\ w_2 = \dfrac{\rho_2(1-v_1-v_3-v_4)}{\rho_2(1-v_1-v_3-v_4)+\rho_1 v_1+\rho_3 v_3+\rho_4 v_4} \text{(Equation S2)} \\ w_3 = \dfrac{\rho_3(1-v_1-v_2-v_4)}{\rho_3(1-v_1-v_2-v_4)+\rho_1 v_1+\rho_2 v_2+\rho_4 v_4} \text{(Equation S3)} \\ w_4 = \dfrac{\rho_4(1-v_1-v_2-v_3)}{\rho_4(1-v_1-v_2-v_3)+\rho_1 v_1+\rho_2 v_2+\rho_3 v_3} \text{(Equation S4)} \\ \rho_{cathode} = \rho_1 v_1+\rho_2 v_2+\rho_3 v_3+\rho_4 v_4 \text{(Equation S5)} \end{cases}$$

Taking typical sulfur cathode as an example, component 3 is carbon nanotube (CNT) and component 4 is sodium carboxymethyl cellulose (CMC). The tap densities and fraction are listed below. Then we can get the functions of density of cathode and density of host material and the plots are listed in Extended Data Figure 1. When selecting CNT (0.19 g cm^{-3}) and $LiCoO_2$ (4 g cm^{-3}) as examples of host materials, the S-cathode densities are 0.8 and 0.49 g cm^{-3}, respectively, by assuming the weight ratio of sulfur: $LiCoO_2$/CNT: CNT: CMC of 0.64:0.16:0.1:0.1.

Table S2.Four components in cathode and the values of physical feature.

Characteristics	Component 1 (sulfur)	Component 2 (Host material)	Component 3 (Conductive agent)	Component 4 (Binder)
Tap density/g cm^{-3}	1.2	ρ_{host}	0.19	0.67
Mass fraction/wt%	0.64	0.16	0.1	0.1
Volume Fraction/%	v_1	v_2	v_3	v_4

The relationship between cathode density and host density is calculated as below:

$$\rho_{cathode} = \frac{\dfrac{1927404837373843465325 1\rho_{host}}{12116075020427264} - \dfrac{7236512259131697129}{594858357280}}{\dfrac{7733687453231300765058\rho_{host}}{593312952031232} + \dfrac{8040569176812996 81}{466074589184}} + \frac{342}{485} \text{(Equation S6)}$$

Electrode porosity

The electrode porosity (ε) is determined from the relation[1]:

$$\varepsilon = 1 - \frac{m_{areal}\left(\dfrac{w_{sulfur}}{\rho_{sulfur}} + \dfrac{w_{host}}{\rho_{host}} + \dfrac{w_{conductive}}{\rho_{conductive}} + \dfrac{w_{CMC}}{\rho_{CMC}} + \dfrac{w_{SBR}}{\rho_{SBR}}\right)}{L} \text{(Equation S7)}$$

where m_{areal} (g cm^{-2})is the mass loading of the electrode except current collector, L(cm) is the electrode thickness, and w (%) and ρ(g cm^{-3}) are the mass fraction and real density of every component, respectively.

Table S3.Real density and mass fraction of every component.

Characteristics	Sulfur	Host		Conductive agent (A-CNT/graphene)	CMC	SBR
		$La_{0.8}Sr_{0.2}MnO_3$	A-CNT			
Real density/g cm^{-3}	2.07	6.5	2.2	2.2	1.6	1.04
Mass fraction/wt%	64.8	15.2	15.2	13	4.2	2.8

Relative density

The densification effect of the electrode could be described by the relative density from the relation[2]:

$$\text{Relative density} = \frac{\rho_{measure}}{\rho_{theory}} \text{(Equation S8)}$$

Where $\rho_{measure}$ (g cm^{-3}) is the electrode density determined by the mass and volume, and ρ_{theory} (g cm^{-3}) is the average real density of the electrode. d_{theory} could be determined from the equation below:

$$\rho_{theory} = \frac{1}{\frac{w_{sulfur}}{\rho_{sulfur}} + \frac{w_{host}}{\rho_{host}} + \frac{w_{conductive}}{\rho_{conductive}} + \frac{w_{CMC}}{\rho_{CMC}} + \frac{w_{SBR}}{\rho_{SBR}}} \quad \text{(Equation S9)}$$

where w(%) and ρ(g cm^{-3}) are the mass fraction and real density of every component, respectively.

Table S4.Porosity of S/$La_{0.8}Sr_{0.2}MnO_3$ and S/A-CNT electrode.

Characteristics	S/$La_{0.8}Sr_{0.2}MnO_3$ electrode		S/A-CNT electrode	
	Bare	Pressed(0.2 MPa)	Bare	Pressed(0.2 MPa)
Porosity (%)	54.5	20.7	77.3	50.9
Relative density (%)	42.9	73.9	22.3	47.0

Calculation of energy density

Volumetric energy density, E_v (Wh L^{-1}), is calculated according to the following equation:

$$E_v = \frac{U \cdot q_{areal}}{d_{cathode}}$$

where U is the voltage at half specific capacity during discharge (V), q_{areal} is the areal capacity of cathode (mAh cm^{-2}), $d_{cathode}$ is the cathode thickness (μm).

Gravimetric energy density, E_g (Wh kg^{-1}), is calculated according to the following equation:

$$E_g = U \cdot q_{cathode}$$

where U is the voltage at half specific capacity during discharge (V), $q_{cathode}$ is the gravimetric capacity of cathode (mAh g^{-1}).

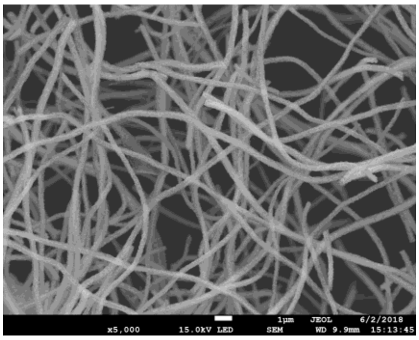

Figure S1.SEM image of $La_{0.8}Sr_{0.2}MnO_3$ nanofibers.

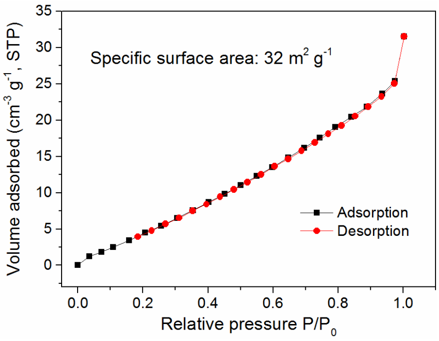

Figure S2.N_2 sorption isotherms of S/$La_{0.8}Sr_{0.2}MnO_3$ composite.

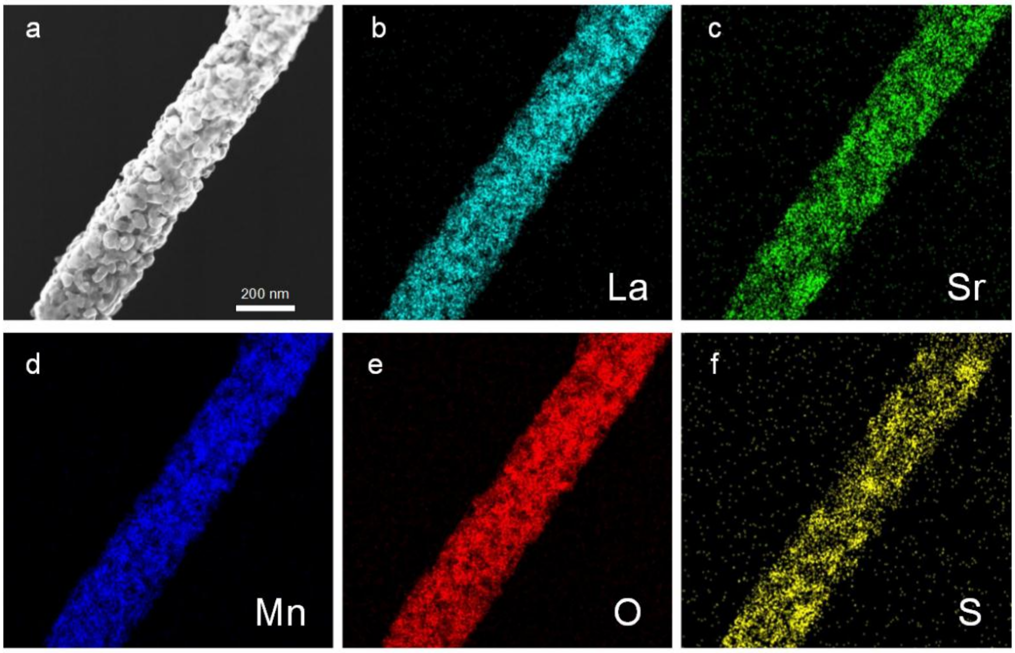

Figure S3.a, STEM image of S/$La_{0.8}Sr_{0.2}MnO_3$ composite and the corresponding EDS of La (**b**), Sr (**c**), Mn (d), O (**e**) and S (**f**) elements.

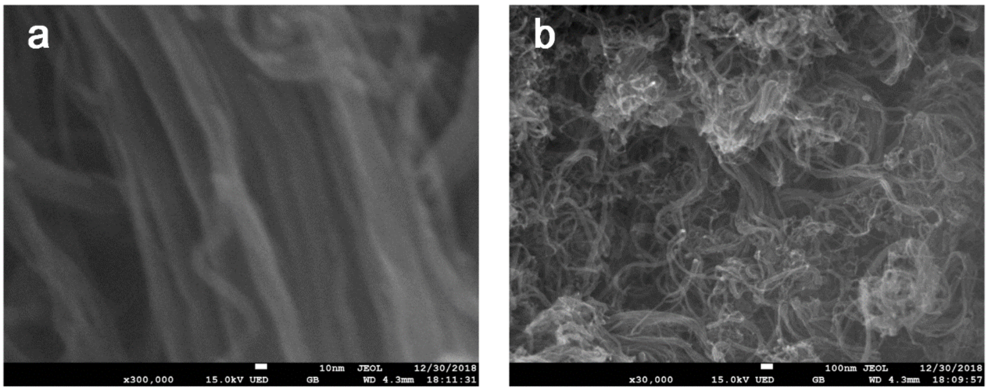

Figure S4.SEM images of A-CNT. a, High resolution. b, Low resolution.

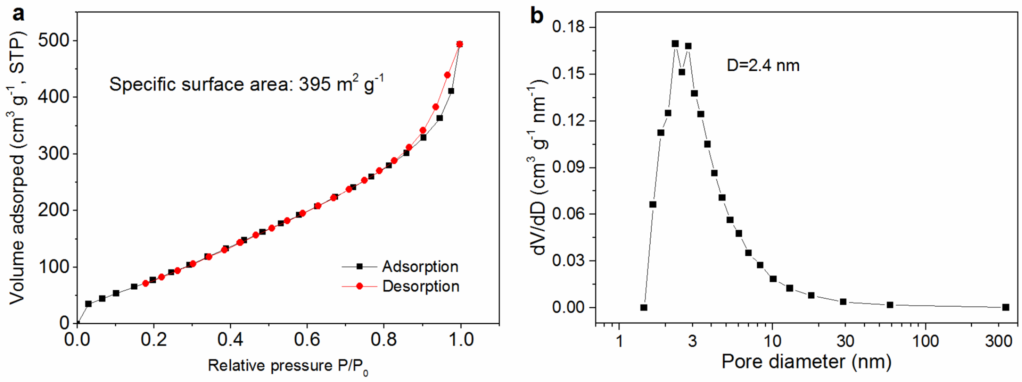

Figure S5.N_2 sorption isotherms. b, pore diameter distributions of A-CNT.

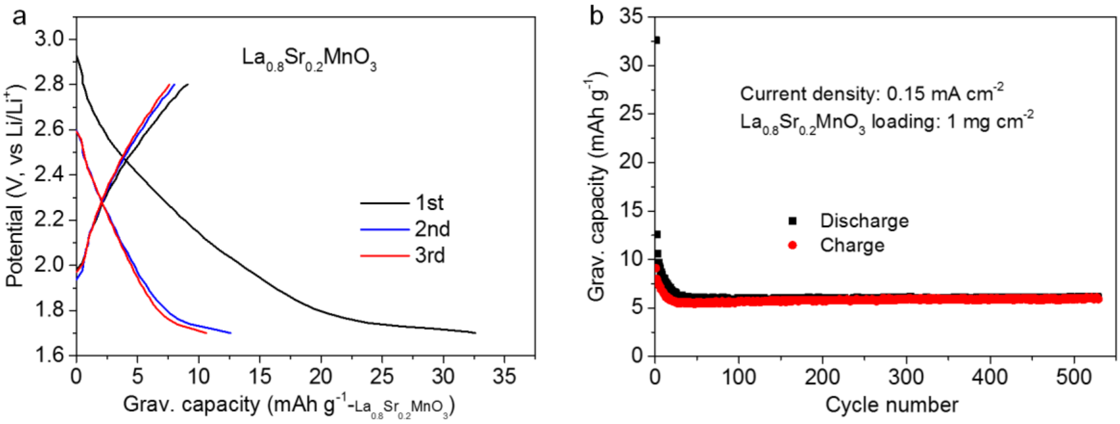

Figure S6.(a) Discharge-charge curves and (b) cycling of $La_{0.8}Sr_{0.2}MnO_3$ electrode at 1.7-2.8 V (vs. Li/Li$^+$).

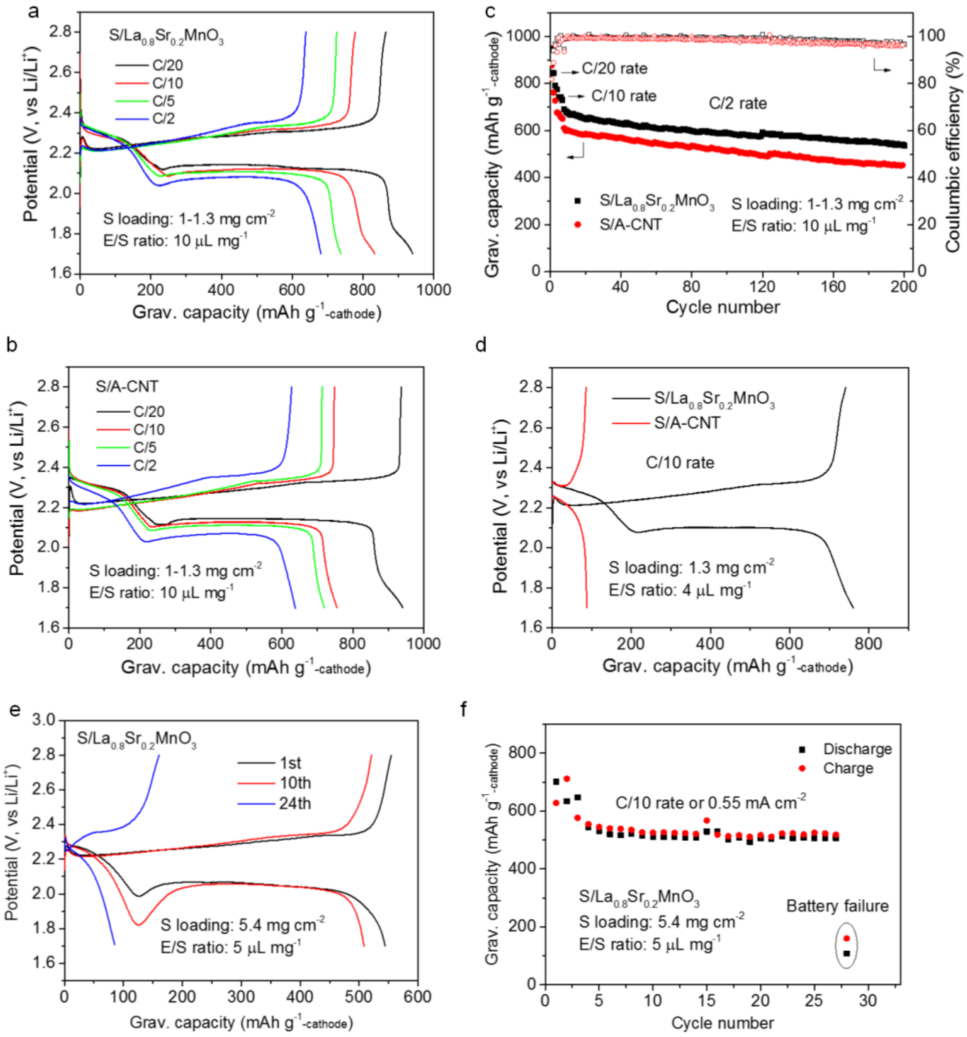

Figure S7. Voltage profiles at various C-rates of (a) S/$La_{0.8}Sr_{0.2}MnO_3$ and (b) S/A-CNT in 10 μL mg^{-1} electrolyte and (c) the cycling stability at c/2 rate with a sulfur loading of 1-1.3 mg cm^{-2}. (d) Voltage profiles of S/$La_{0.8}Sr_{0.2}MnO_3$ and S/A-CNT in 4 μL mg^{-1} electrolyte. (e) Voltage profiles of S/$La_{0.8}Sr_{0.2}MnO_3$ and (f) the cycling stability in 5 μL mg^{-1} electrolyte with a sulfur loading of 5.4 mg cm^{-2}.

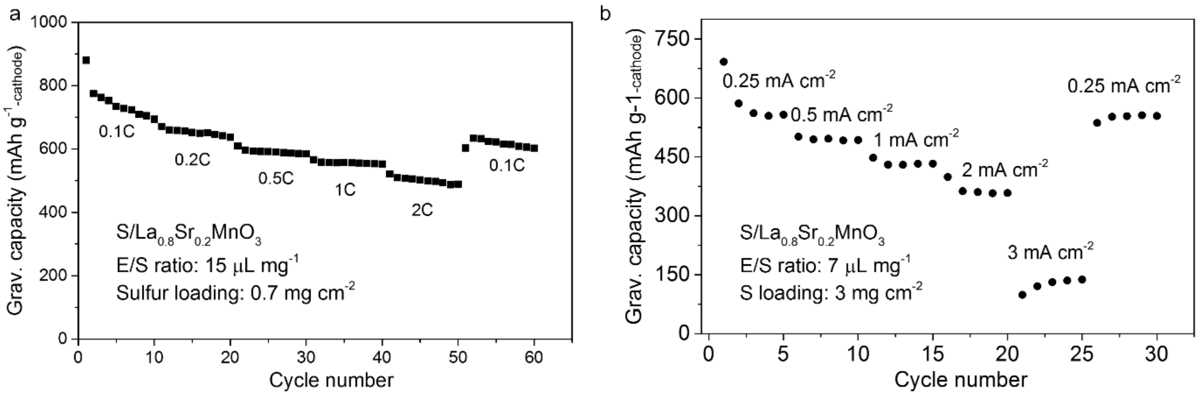

Figure S8. Rate performance of S/$La_{0.8}Sr_{0.2}MnO_3$ cathode.

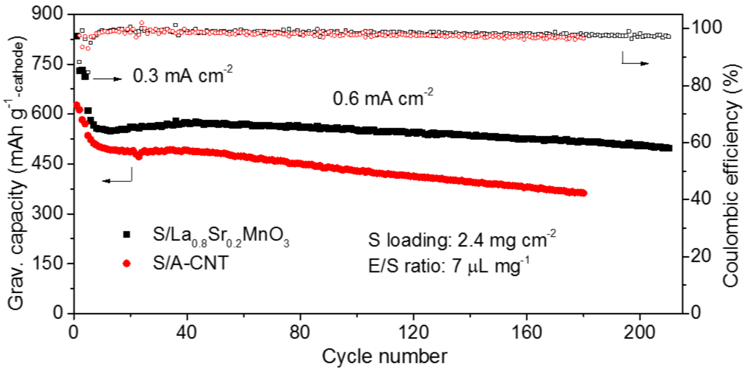

Figure S9. Cycling of S/$La_{0.8}Sr_{0.2}MnO_3$ and S/A-CNT cathodes at 2.4 mg cm^{-2} sulfur loading.

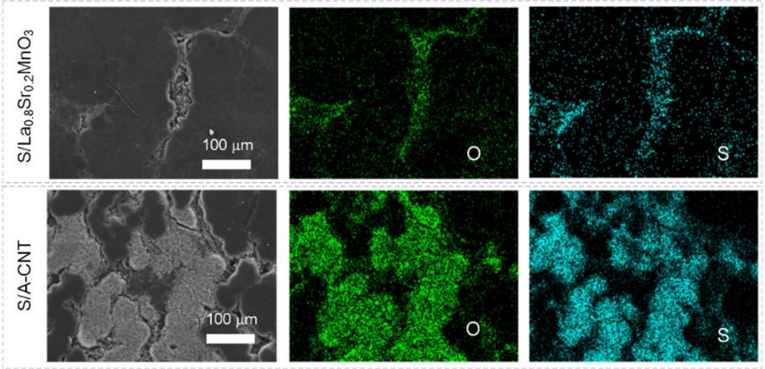

Figure S10. SEM images of lithium anode after 100 cycles at C/10 rate.

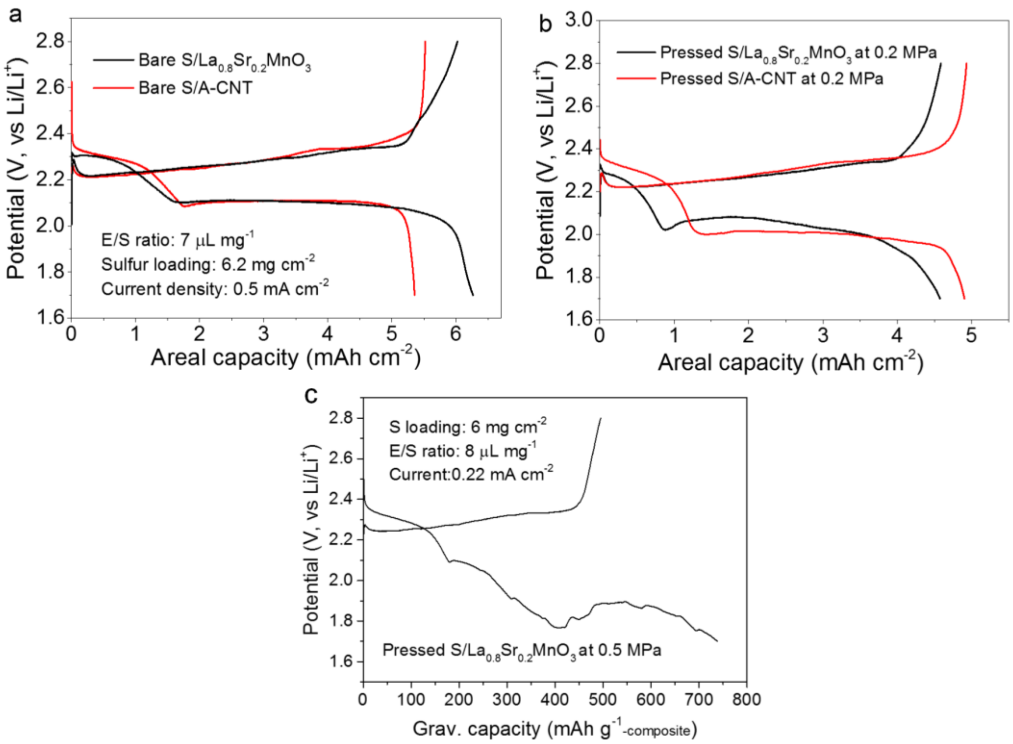

Figure S11. Discharge-charge curves of the S/$La_{0.8}Sr_{0.2}MnO_3$ and S/A-CNT cathodes. (a) Bare cathodes. (b) Pressed cathodes at 0.2 MPa. (c) S/$La_{0.8}Sr_{0.2}MnO_3$ cathode pressed at 0.5 MPa.

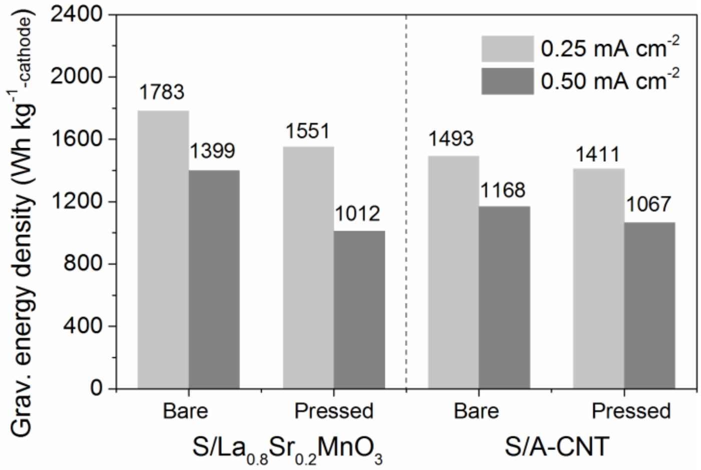

Figure S12. Comparison of the gravimetric energy density of S/La$_{0.8}$Sr$_{0.2}$MnO$_3$ and S/A-CNT cathodes before and after pressing. Cathode refers to the sulfur-based composite, conductive agent and binder, without current collector.

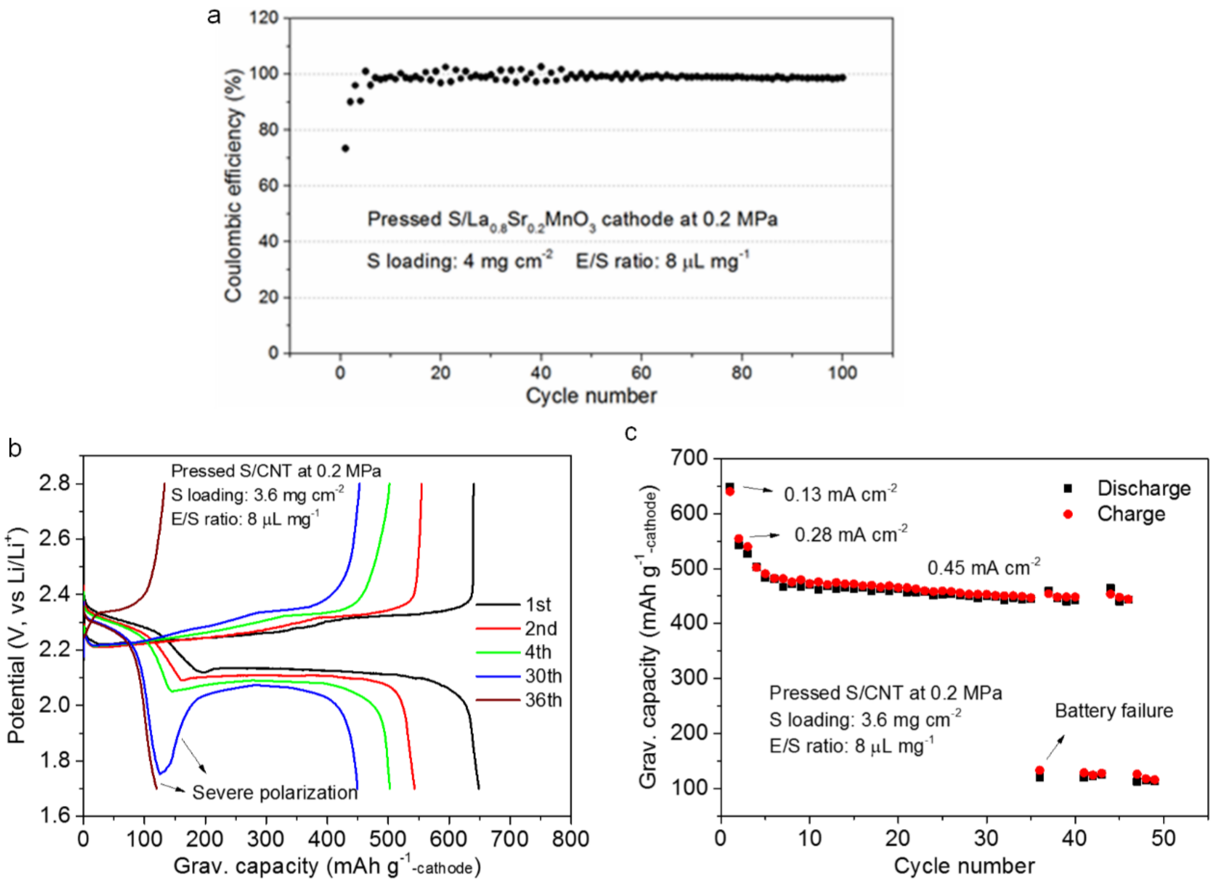

Figure S13. (a) Coulombic efficiency of S/La$_{0.8}$Sr$_{0.2}$MnO$_3$cathode pressed at 0.2 MPa. (b) and (c) are the voltage profiles and cycling of pressed S/A-CNT cathode, respectively.

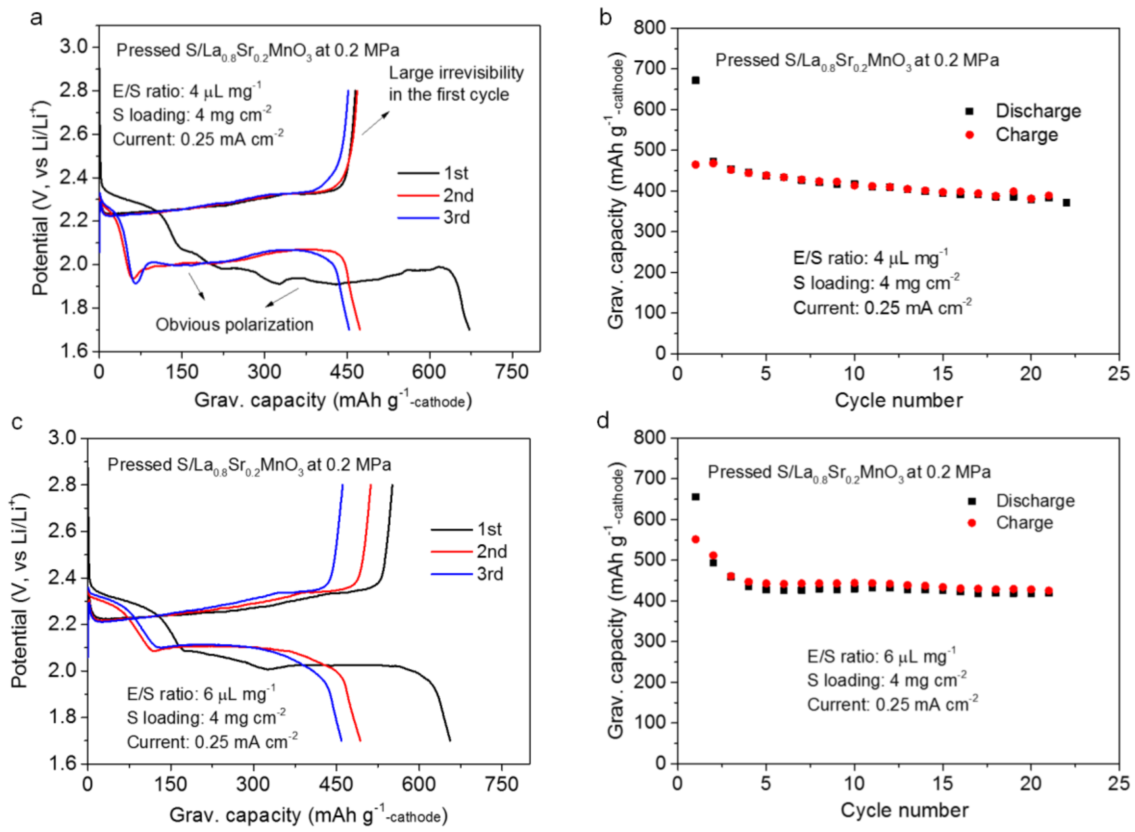

Figure S14.(a)and (b) are voltage profiles and cycling stability of pressed S/$La_{0.8}Sr_{0.2}MnO_3$cathode at a E/S ratio of 4 μL mg^{-1}, respectively. (c)and (d) are voltage profiles and cycling stability of pressed S/$La_{0.8}Sr_{0.2}MnO_3$cathode at a E/S ratio of 6 μL mg^{-1}, respectively. Note the cells are still cycling in our lab.

Table S5. Summary of volumetric capacity reported in recent works.

No.	Sulfur host	Current density	Areal S loading mg/cm^2	Vol. capacity mAh/cm^3	Vol. energy density Wh/L	Freestanding or not	Ref
1	Graphene	0.5C		233	489.3	Not	[3]
2	Super P/MWCNT	0.1C	1.3-1.5	530	1113	Not	[4]
3	Graphene/ graphite	0.1C	3.6	745	1527	Not	[5]
4	Graphene/CNT	0.5C	4.7	406	850	Yes	[6]
5	Carbon spheres /MWCNT	0.05C	5	1106	2323	Yes	[7]
6	Carbon fiber	0.1C	30.7	1201	2522	Yes	[8]
7	Carbon network	0.1C	10.9	867	1777	Not	[9]
8	Graphene microspheres	0.03C	2.5	160	336	Yes	[10]
9	CNT	0.25C	6.3	200	420	Yes	[11]
10	CNF	1.5 mA cm^{-2}	18.1	335	704	Yes	[12]
11	CNF		2	240	504	Yes	[13]
12	CNT		7	260	546	Not	[14]
13	PAN		6.7	270	486	Not	[15]
14	Carbon sphere/graphene		3.9	320	672	Yes	[16]
15	Graphene		2.5	510	1071	Yes	[17]
16	CNT	0.1C	4.5	570	1169	Yes	[18]
17	TiO_2/Graphene		3.2	640	1344	Yes	[19]
18	CNF		10.8	380	779	Yes	[20]
19	Porous carbon polyhedrons		6.4	830	1702	Yes	[21]
20	Graphene-PEDOT:PSS	0.2C	2	1261	2648	Yes	[22]
21	Porous carbon nanospheres	0.05C	5	917	1926	Not	[23]
22	Porous g-C_3N_4	0.05C	5.2	490	1029	Not	[24]
23	Ketjenblack	0.1C	4	650	1365	Not	[25]
24	Graphene/VS_2	0.1C	2.56	1182.1	2482	Yes	[26]
25	A-CNT	0.1C	1.2	1116	2344	Not	[27]
26	CNF	0.1C	2.32	1103	2317	Yes	[28]
27	Mo_6S_8	0.5 mA cm^{-2}	6.9	477.4	1002.5	not	[29]
28	Co_9S_8	0.2C	4.5	846.2	1775	not	[30]
This work	Pressed LSMO	0.25 mA cm^{-2}	6.2	1330	2726.5	not	
		0.5 mA cm^{-2}	6.2	867	1778.5	not	
		0.25 mA cm^{-2}	4	1243	2610.3	not	
		0.5 mA cm^{-2}	4	935	1917	not	
	Pressed A-CNT	0.25 mA cm^{-2}	6.2	623	1308.3	not	
		0.5 mA cm^{-2}	6.2	492	984	not	
	Bare A-CNT	0.25 mA cm^{-2}	6.2	328	689	not	
		0.5 mA cm^{-2}	6.2	257	540	not	
	Pressed LSMO	0.25 mA cm^{-2}	6.2	851	1787	not	
		0.5 mA cm^{-2}	6.2	668	1403	not	

Randles-Sevcik euqtion[31]:

$$i_p = (2.69 \times 10^5) n^{3/2} A D_0^{1/2} v^{1/2} C_0^* \text{ (25 °C)} \quad \text{(Equation S10)}$$

wherei_p is the peak current (A), *n* is the charge transfer number, *A* is the active electrode area (cm^2), D_0 is the lithium ion diffusion coefficient ($cm^2\ s^{-1}$), *v* is the scan rate ($V\ s^{-1}$), and C_0^* is the concentration ofLiPS($mol\ cm^{-3}$).

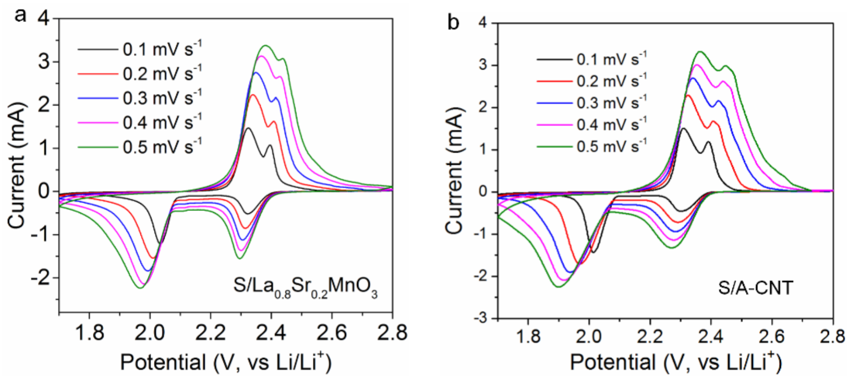

Figure S15.CV curves of S/La$_{0.8}$Sr$_{0.2}$MnO$_3$ (a) and S/A-CNT(b) composite at scan rates ranging from 0.1 to 0.5 mV s^{-1}.

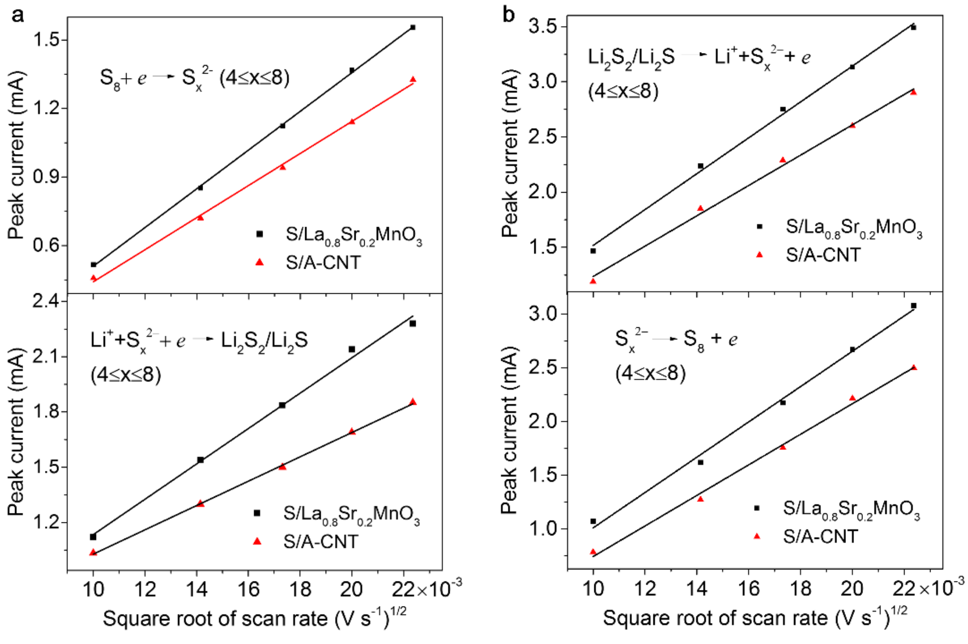

Figure S16. Relationships of peak current and square root of scan rate for different redox peaks. Stepwise reduction (a) of sulfur: S_8+*e*→S_x^{2-}(4≤x8), Li^++S_x^{2-}+*e*→Li_2S_2/Li_2S (4≤x8) and Stepwise oxidation (b) of lithium sulfide: Li_2S_2/Li_2S→Li^++S_x^{2-}+*e* (4≤x8), S_x^{2-}→S_8+*e* (4≤x8).

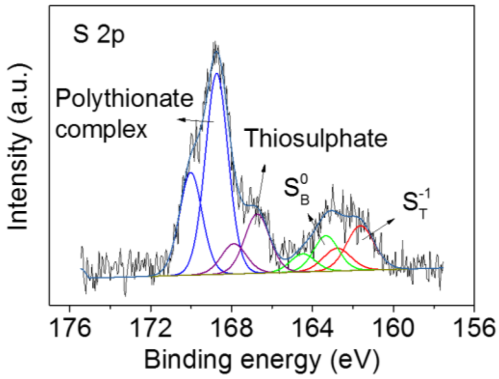

Figure S17. Chemical interactions between Li_2S_4 and $La_{0.8}Sr_{0.2}MnO_3$ characterized by XPS. S 2p core level of Li_2S_4/ $La_{0.8}Sr_{0.2}MnO_3$.

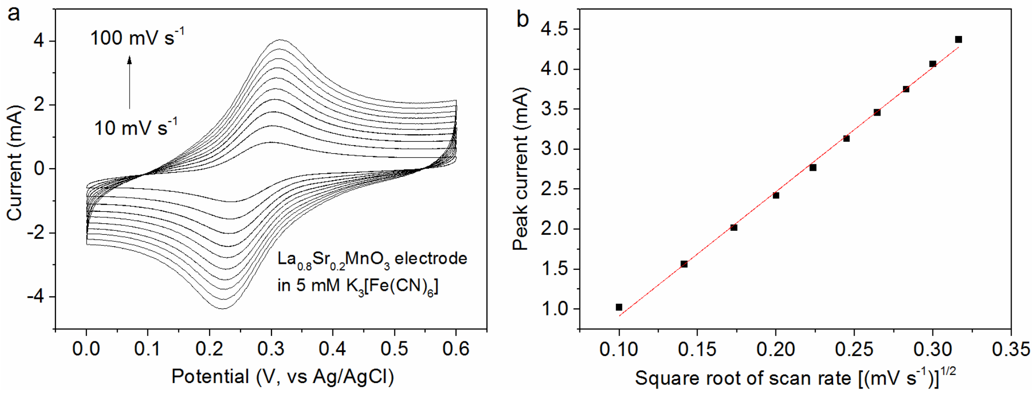

Figure S18.(a) CVs of A-CNT electrode in 5 mM$K_3[Fe(CN)_6]$ electrolyte at various scan rates. (b) the linear relationship between square root of scan rate and peak current.

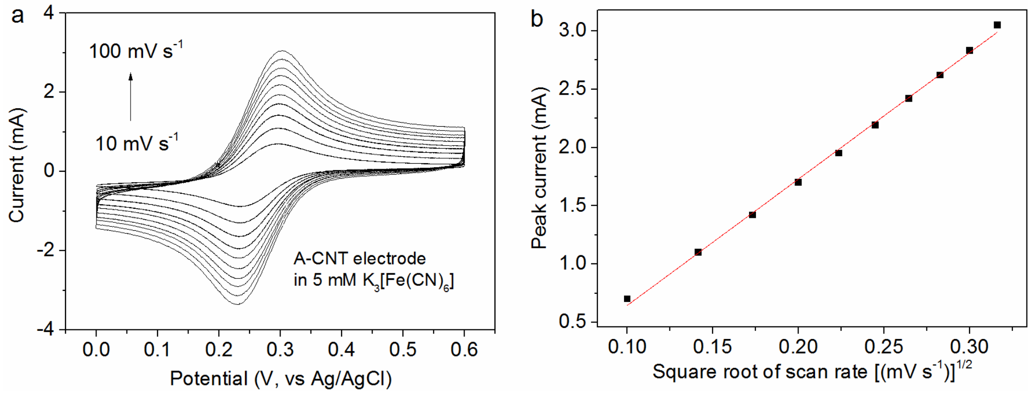

Figure S19.(a) CVs of A-CNT electrode in 5 mM$K_3[Fe(CN)_6]$ electrolyte at various scan rates. (b) The linear relationship between square root of scan rate and peak current.

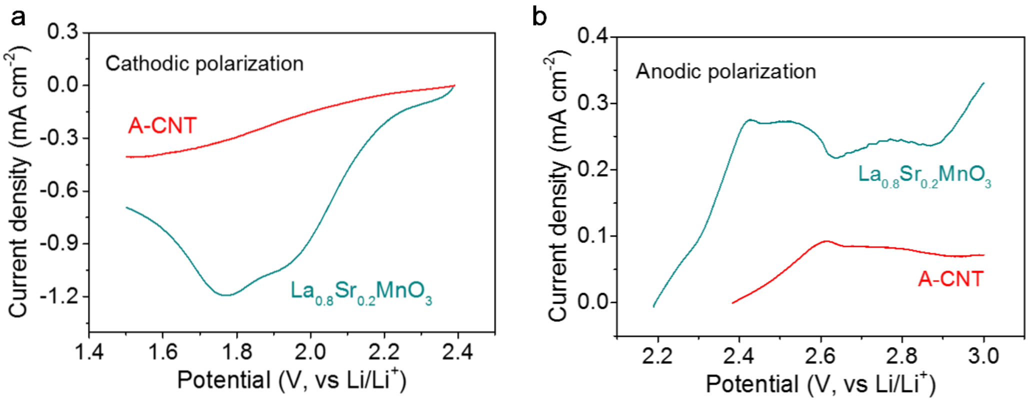

Figure S20. *J-V* curves showing the catalytic performance of $La_{0.8}Sr_{0.2}MnO_3$ in comparison to A-CNT electrode. (a) Cathodic polarization. (b) Anodic polorization.

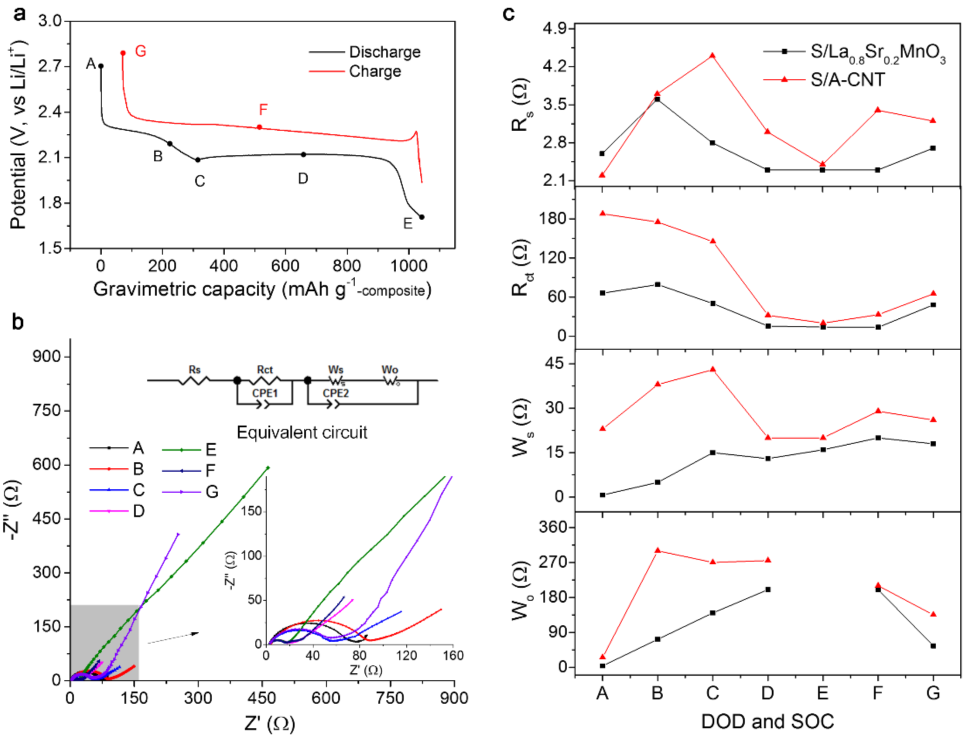

Figure S21. Interface electrochemistry of LiPS by *in-situ* EIS test.(a) Typical discharge-charge curves of S/$La_{0.8}Sr_{0.2}MnO_3$ for EIS test. (b)Nyquist plots of S/$La_{0.8}Sr_{0.2}MnO_3$ at various DOD and SOC. In the equivalent circuit, R_s is the short intercept, R_{ct}//CPE1 and W_s//CPE2 are used for modeling the depressed semicircle, and W_o//CPE2 is used for modeling the slope line. A CPE is selected instead of capacitor due to the inhomogeneity and roughness of the electrode. (c) Plots of R_s, R_{ct}, W_s and W_o against DOD and DOC. Note the W_o at point E is not included because it's too hard to determine the accurate value.

[1] C. Heubner, A. Nickol, J. Seeba, S. Reuber, N. Junker, M. Wolter, M. Schneider, A. Michaelis, *J. Power Sources* **2019**, *419*, 119.
[2] C. Laberty-Robert, F. Ansart, C. Deloget, M. Gaudon, A. Rousset, *Ceram. Int.* **2003**, *29*, 151.
[3] C. Zhang, D.-H. Liu, W. Lv, D.-W. Wang, W. Wei, G.-M. Zhou, S. Wang, F. Li, B.-H. Li, F. Kang, Q.-H. Yang, *Nanoscale* **2015**, *7*, 5592.
[4] K. R. Kim, K.-S. Lee, C.-Y. Ahn, S.-H. Yu, Y.-E. Sung, *Sci. Rep.* **2016**, *6*, 32433.
[5] G. Babu, L. M. Reddy Arava, *RSC Advances* **2015**, *5*, 47621.
[6] P.-Y. Zhai, J.-Q. Huang, L. Zhu, J.-L. Shi, W. Zhu, Q. Zhang, *Carbon* **2017**, *111*, 493.
[7] C. Hu, C. Kirk, J. Silvestre-Albero, F. Rodríguez-Reinoso, M. J. Biggs, *J. Mater. Chem. A* **2017**, *5*, 19924.
[8] S.-H. Chung, C.-H. Chang, A. Manthiram, *ACS Nano* **2016**, *10*, 10462.
[9] X. Yu, J. Deng, R. Lv, Z.-H. Huang, B. Li, F. Kang, *Energy Storage Mater.* **2018**, *20*, 14.
[10] J.-L. Shi, H.-J. Peng, L. Zhu, W. Zhu, Q. Zhang, *Carbon* **2015**, *92*, 96.
[11] Z. Yuan, H.-J. Peng, J.-Q. Huang, X.-Y. Liu, D.-W. Wang, X.-B. Cheng, Q. Zhang, *Adv. Funct. Mater.* **2014**,*24*, 6105.
[12] L. Qie, C. Zu, A. Manthiram, *Adv. Energy Mater.* **2016**, *6*, 1502459.
[13] Z. Zhang, Q. Li, K. Zhang, W. Chen, Y. Lai, J. Li, *J. Power Sources* **2015**, *290*, 159.
[14] X.-B. Cheng, H.-J. Peng, J.-Q. Huang, L. Zhu, S.-H. Yang, Y. Liu, H.-W. Zhang, W. Zhu, F. Wei, Q. Zhang, *J. Power Sources* **2014**, *261*, 264.
[15] J.-S. Kim, T. H. Hwang, B. G. Kim, J. Min, J. W. Choi, *Adv. Funct. Mater.* **2014**, *24*, 5359.
[16] G. Zhou, Y. Zhao, A. Manthiram, *Adv. Energy Mater.* **2015**, *5*, 1402263.
[17] F. Li, G. Zhou, S. Pei, L. Li, D. W. Wang, S. Wang, K. Huang, L. C. Yin, H. M. Cheng, *Adv. Mater.* **2014**, *26*, 625.
[18] X. Fang, W. Weng, J. Ren, H. Peng, *Adv. Mater.* **2016**, *28*, 491.
[19] G. Zhou, Y. Zhao, C. Zu, A. Manthiram, *Nano Energy* **2015**, *12*, 240.
[20] Z. Li, J. T. Zhang, Y. M. Chen, J. Li, X. W. Lou, *Nat. Commun.* **2015**, *6*, 8850.
[21] Y. Liu, G. Li, J. Fu, Z. Chen, X. Peng, *Angew. Chem. Int. Ed.* **2017**, *56*, 6176.
[22] P. Xiao, F. Bu, G. Yang, Y. Zhang, Y. Xu, *Adv. Mater.* **2017**, *29*, 1703324.
[23] C. Hu, C. Kirk, Q. Cai, C. Cuadrado-Collados, J. Silvestre-Albero, F. Rodríguez-Reinoso, M. J. Biggs, *Adv. Energy Mater.* **2017**, *7*, 1701082.
[24] Q. Pang, X. Liang, C. Y. Kwok, J. Kulisch, L. F. Nazar, *Adv. Energy Mater.* **2017**, *7*, 1601630.
[25] D. Lu, Q. Li, J. Liu, J. Zheng, Y. Wang, S. Ferrara, J. Xiao, J.-G. Zhang, J. Liu, *ACS Appl. Mater. Interfaces* **2018**, *10*, 23094.
[26] Z. Cheng, Z. Xiao, H. Pan, S. Wang, R. Wang, *Adv. Energy Mater.* **2018**, *8*, 1702337.
[27] X. B. Cheng, J. Q. Huang, Q. Zhang, H. J. Peng, M. Q. Zhao, F. Wei, *Nano Energy* **2014**, *4*, 65.
[28] H. Pan, J. Chen, R. Cao, V. Murugesan, N. N. Rajput, K. S. Han, K. Persson, L. Estevez, M. H. Engelhard, J.-G. Zhang, K. T. Mueller, Y. Cui, Y. Shao, J. Liu, *Nat. Energy* **2017**, *2*, 813.
[29] W. Xue, Z. Shi, L. Suo, C. Wang, Z. Wang, H. Wang, K. P. So, A. Maurano, D. Yu, Y. Chen, L. Qie, Z. Zhu, G. Xu, J. Kong, J. Li, *Nat. Energy* **2019**, *4*, 374.
[30] Q. Pang, D. Kundu, L. F. Nazar, *Mater. Horiz.* **2016**, *3*, 130.
[31] X. Y. Tao, J. G. Wang, C. Liu, H. T. Wang, H. B. Yao, G. Y. Zheng, Z. W. Seh, Q. X. Cai, W. Y. Li, G. M. Zhou, C. X. Zu, Y. Cui, *Nat. Commun.* **2016**, *7*, 11203.
